# Human Islet Amyloid Polypeptide Fibril Binding to Catalase: A Transmission Electron Microscopy and Microplate Study

**DOI:** 10.1100/tsw.2010.73

**Published:** 2010-05-18

**Authors:** Nathaniel G. N. Milton, J. Robin Harris

**Affiliations:** ^1^ Department of Human and Health Sciences, School of Life Sciences, University of Westminster, London, UK; ^2^ Institute of Zoology, University of Mainz, Germany; ^3^ Institute of Cell and Molecular Biosciences, University of Newcastle, Newcastle-upon-Tyne, UK

**Keywords:** catalase, amylin, islet amyloid polypeptide, amyloid, prion protein, diabetes, noninsulin-dependent diabetes mellitus (NIDDM; Type II diabetes), fibril

## Abstract

The diabetes-associated human islet amyloid polypeptide (IAPP) is a 37-amino-acid peptide that forms fibrils *in vitro* and *in vivo*. Human IAPP fibrils are toxic in a similar manner to Alzheimer's amyloid-β (Aβ) and prion protein (PrP) fibrils. Previous studies have shown that catalase binds to Aβ fibrils and appears to recognize a region containing the Gly-Ala-Ile-Ile sequence that is similar to the Gly-Ala-Ile-Leu sequence found in human IAPP residues 24-27. This study presents a transmission electron microscopy (TEM)—based analysis of fibril formation and the binding of human erythrocyte catalase to IAPP fibrils. The results show that human IAPP 1-37, 8-37, and 20-29 peptides form fibrils with diverse and polymorphic structures. All three forms of IAPP bound catalase, and complexes of IAPP 1-37 or 8-37 with catalase were identified by immunoassay. The binding of biotinylated IAPP to catalase was high affinity with a K_D_ of 0.77nM, and could be inhibited by either human or rat IAPP 1-37 and 8-37 forms. Fibrils formed by the PrP 118-135 peptide with a Gly-Ala-Val-Val sequence also bound catalase. These results suggest that catalase recognizes a Gly-Ala-Ile-Leu—like sequence in amyloid fibril-forming peptides. For IAPP 1-37 and 8-37, the catalase binding was primarily directed towards fibrillar rather than ribbon-like structures, suggesting differences in the accessibility of the human IAPP 24-27 Gly-Ala-Ile-Leu region. This suggests that catalase may be able to discriminate between different structural forms of IAPP fibrils. The ability of catalase to bind IAPP, Aβ, and PrP fibrils demonstrates the presence of similar accessible structural motifs that may be targets for antiamyloid therapeutic development.

### INTRODUCTION

The islet amyloid polypeptide (IAPP), or amylin peptide, is a hallmark of Type II diabetes mellitus (DM) pathology where it forms amyloid deposits[1,2,3]. The IAPP readily aggregates both *in vitro*[4,5,6] and *in*

*vivo*[7] to form amyloid fibrils that can be readily studied using electron microscopy[8]. Fibrillogenesis of IAPP can be studied by transmission electron microscopy (TEM) and atomic force microscopy (AFM). Polymorphism of IAPP fibrils, Alzheimer's amyloid-β (Aβ) fibrils, and the prion protein (PrP) fibrils is influenced by the environment in which the fibrillogenesis occurs[9,10,11,12,13]. Aggregated forms of IAPP, Aβ, and PrP all act via similar mechanisms to cause neurotoxicity[14,15].

The antioxidant enzyme catalase is also expressed in the pancreas[16] and partially protects pancreatic islet cell lines from human IAPP toxicity[17]. Catalase contains a heme group[18], an NADPH binding site[19], and exists as a tetramer[20,21,22,23]. Previous studies have demonstrated Aβ binding to catalase[24] and suggested that the catalase 400-409 region contained an Aβ binding domain[25]. The catalase 400-409 region is within the wrapping loop that may play a role in the formation of the tetramer structure[26] and should be accessible to Aβ or related peptides.

Type II DM is associated with IAPP plaque deposition in the pancreas[1] and has also been linked to decreased catalase levels[27]. The pancreatic cell death associated with DM has been linked to both IAPP toxicity[28] and increased susceptibility to oxidative stress[29]. Low levels of circulating catalase are associated with increased pancreatic damage in animal models of DM[30]. There has also been a suggestion that catalase levels in the pancreas are up-regulated in Type II DM[31], possibly as a protective response to the condition. Human IAPP also has central neurohormonal actions that play a role in obesity[32] and has been shown to have neurotoxicity[15].

In a recent study, we demonstrated that the Aβ 29-32 region was essential for catalase binding to Aβ fibrils[11]. The Gly-Ala-Ile-Ile sequence of the Aβ 29-32 region is similar to the human IAPP 24-27 and the human PrP 119-122 sequences[11], raising the possibility that catalase may also directly interact with IAPP or PrP fibrils.

The present study was undertaken to investigate the fibrillogenesis and polymorphism of human IAPP peptide fragments, derived from the naturally occurring human IAPP 1-37 amino acid sequence produced *in vitro* by 24-h incubation at 37°C. The binding of catalase to the varying preformed human IAPP fibrils was also studied to determine whether catalase can bind IAPP and discriminate between different IAPP fibril polymorphic forms. A comparison with catalase binding to Aβ fibrils and human PrP fragment fibrils was also performed.

### MATERIALS AND METHODS

Human or rat IAPP (1-37, 8-37, and 20-29), Aβ peptides (1-42, 17-40, and 31-35), PrP peptide (118-135), pepstatin A peptide (PepA), and corticotrophin releasing factor-41 peptide were purchased from either American Peptides, Bachem, or Sigma-Aldrich. N-terminal biotinyl human IAPP 1-37 was purchased from Alpha Diagnostic International. Biotinyl-LHRH was obtained from Bachem. Monoclonal antiamylin antibody ND-1 was obtained from Insight Biotechnology. Anticatalase antibody was purchased from Calbiochem. Human erythrocyte catalase (HEC), alkaline phosphatase antirabbit IgG conjugate, and alkaline phosphatase polymer-streptavidin conjugate were purchased from Sigma-Aldrich.

#### *In vitro* Fibrillogenesis and Catalase Binding to Fibrils

Fibril formation from the human IAPP, Aβ, PrP, and PepA peptides was induced at a concentration of 1.0 mg/ml after dissolving in distilled water or 20 mM Tris-HCl pH 7.4, followed by incubation at 37°C for 24 h, with constant oscillation[11,33,34]. Following incubation, aliquots were then taken from the test samples, now containing preformed fibrils, with subsequent addition of HEC (0.05 mg/ml) and subsequent incubation for periods of 2 h at room temperature (22°C), to allow attachment of the catalase. Samples containing preformed IAPP 1-37 or Aβ 17-40 fibrils were also incubated with catalase (0.05 mg/ml), with and without Aβ 31-35 (1 mg/ml).

### Specimen Preparation and TEM Study

Negatively stained specimens were prepared on continuous carbon support films using the single-droplet procedure, with 2% w/v aqueous uranyl acetate[34,35]. The TEM study was performed using a Zeiss EM901, a Philips CM12, and a FEI Tecnai 12. Images were recorded photographically on Kodak type 4489 and SO163 electron image film, and digitally with a column-mounted Mega View III CCD camera. Photographic negatives were scanned using an Epson Perfection 1640SU, as ~3 MB .tif files with an image resolution of at least 600 dpi.

### Immunoassay for Catalase-IAPP Complexes

ELISA plates were coated with a mouse antiamylin monoclonal (ND1; 1 µg/ml) in 50 mM carbonate buffer pH 9.6 and unoccupied sites blocked with 5% (w/v) Marvel. Human IAPP 1-37, rat IAPP 1-37, human IAPP 8-37, rat IAPP 8-37, or human IAPP 20-29 peptides (10 µg/ml) were incubated with catalase (0.05 mg/ml) for 2 h at room temperature (22°C) in 50 mM Tris-HCl buffer (pH 7.5). Control samples containing IAPP peptides alone, IAPP peptides plus bovine serum albumin, catalase alone, and catalase plus unrelated peptide (corticotrophin releasing factor-41) were also prepared and incubated for 2 h at room temperature (22°C) in 50 mM Tris-HCl buffer (pH 7.5). Samples were then added to antiamylin-coated plates and incubated for 2 h at room temperature (22°C). After washing with assay buffer to remove unbound material, polyclonal rabbit anticatalase antibody was added and incubated for 2 h at room temperature (22°C). After washing with assay buffer to remove unbound material, an alkaline phosphatase antirabbit IgG conjugate was added and incubated for 2 h at room temperature (22°C). After washing to remove unbound material, p-nitrophenylphosphate substrate was added and absorbance at 405 nm was determined[36]. Control values from wells containing buffer alone in place of samples were determined (n = 6). All samples were tested in groups of six and the experiments repeated on at least three occasions with different batches of antiamylin-coated plates.

### Catalase Binding to Labeled IAPP

ELISA plates were coated with catalase (1 µg/ml) in 50 mM carbonate buffer and unoccupied sites blocked with 5% (w/v) Marvel. For competition studies, molar concentrations were calculated based on the monomeric molecular weight of each peptide. Peptides for these studies were dissolved in DMSO and incubated in 50 mM Tris-HCl buffer (pH 7.5), a method that previous studies have suggested limited fibril formation by IAPP peptides[37]. Biotinyl human IAPP (200 pM) was added to catalase-coated plates and incubated alone or with unlabeled human IAPP, PrP, and Aβ peptides (10 µM) in 50 mM Tris-HCl buffer (pH 7.5) at 37°C for 4 h. In some experiments, catalase-coated plates were preincubated with unlabeled human IAPP, PrP, and Aβ peptides (10 µM) for 4 h at 37°C prior to washing with assay buffer to remove unbound material and then incubation with biotinyl human IAPP (200 pM) at 37°C for 4 h. After washing with assay buffer to remove unbound material, an alkaline phosphatase polymer-streptavidin conjugate was added and incubated at 37°C for 2 h. After washing to remove unbound material, p-nitrophenylphosphate substrate was added and absorbance at 405 nm was determined[24]. Control values from wells containing buffer alone in place of samples were determined (n = 6). All samples were tested in groups of six and the experiments repeated on at least three occasions with different batches of catalase-coated plates.

The affinity constant was determined by incubating catalase-coated plates with biotinyl human IAPP 1-37 (200 pM) plus unlabeled IAPP 1-37 over a range of concentrations (0–100 nM) and detection of bound peptides by ELISA. Scatchard analysis[24,38] was performed using the following equation: $A_0 / (A_0 - A) = 1 + K_D / a_0$ and plotting $v[(A_0 - A) / A_0]$ against $v / a$, where $A_0$ = absorbance in absence of unlabeled peptide, A = absorbance in presence of unlabeled peptide, $a_0$ = total concentration of unlabeled peptide, and a = concentration of unlabeled peptide added. The $K_D$ was equal to - 1 / slope of v against v / a.

### Catalase Binding to Unlabeled IAPP

ELISA plates were coated with human IAPP 1-37 (1 µg/ml) in 50 mM carbonate buffer and unoccupied sites blocked with 5% (w/v) Marvel. Catalase (500 nM) was added to human IAPP–coated plates and incubated alone or with unlabeled human IAPP, Aβ, or PrP peptides (10 µM) in 50 mM Tris-HCl buffer (pH 7.5) at 37°C for 4 h. After washing with assay buffer to remove unbound material, polyclonal rabbit anticatalase antibody was added and incubated for 2 h at room temperature (22°C). After washing with assay buffer to remove unbound material, an alkaline phosphatase antirabbit IgG conjugate was added and incubated for 2 h at room temperature (22°C). After washing to remove unbound material, p-nitrophenylphosphate substrate was added and absorbance at 405 nm was determined[24]. Control values from wells containing buffer alone in place of samples were determined (n = 6). All samples were tested in groups of six and the experiments repeated on at least three occasions with different batches of IAPP-coated plates.

### Amyloid Peptide Binding to Labeled IAPP

ELISA plates were coated with either human IAPP 1-37, human IAPP 8-37, human IAPP 20-29, rat IAPP 1-37, Aβ 1-42, PrP 118-135, or PepA peptides (1 µg/ml) in 50 mM carbonate buffer and unoccupied sites blocked with 5% (w/v) Marvel. Biotinyl human IAPP (200 pM), a control peptide (biotinyl-LHRH), or unlabeled human IAPP 1-37 was added to coated plates and incubated in 50 mM Tris-HCl buffer (pH 7.5) for 4 h at 37°C. After washing with assay buffer to remove unbound material, an alkaline phosphatase polymer-streptavidin conjugate was added and incubated at 37°C for 2 h. After washing to remove unbound material, p-nitrophenylphosphate substrate was added and absorbance at 405 nm was determined[24]. Control values from wells containing buffer alone in place of samples were determined (n = 6). All samples were tested in groups of six and the experiments repeated on at least three occasions with different batches of catalase-coated plates.

## RESULTS

### TEM of IAPP Fibrils

Fibril, ribbon-like, and helical twist structures in IAPP 1-37 have been described[39] and we have used the same terms to describe the observed fibrils in this study. A schematic diagram of fibrils, fibrils with twists, ribbon-like structures, ribbons with helical twists, and sheet-like structures is shown in Fig. 1. For clarity, the number of fibrils making up a ribbon or sheet-like structure is in a ratio of 1:3 for the schematic; however, in reality, the number of fibrils covers a wide range. The sheet-like structures described are similar to those we have previously described for Aβ 17-28[11], and we have previously suggested that Aβ ribbons are aggregates of fibrils and may represent intermediates between fibril and sheet forms; similar observations may also apply to IAPP fibrils, ribbons, and sheets. Fibrils were formed from human IAPP 1-37 (Fig. 1A), 8-37 (Fig. 1B), and 20-29 (Figs. 1C and 1D). The fibrils showed polymorphic forms with IAPP 1-37 (Fig. 1A) and 8-37 (Fig. 1B) containing a mixture of fibrils and ribbon-like structures with helical twists in these ribbons. The IAPP 20-29 (Fig. 1C) also showed polymorphic forms with some thin fibrils, and some much larger tubular and sheet-like structures. The larger IAPP 20-29 tubular and sheet-like structures (Fig. 1D) appeared to be formed by thin fibrils that had self-assembled in a parallel side-by-side orientation as indicated in the schematic diagram.

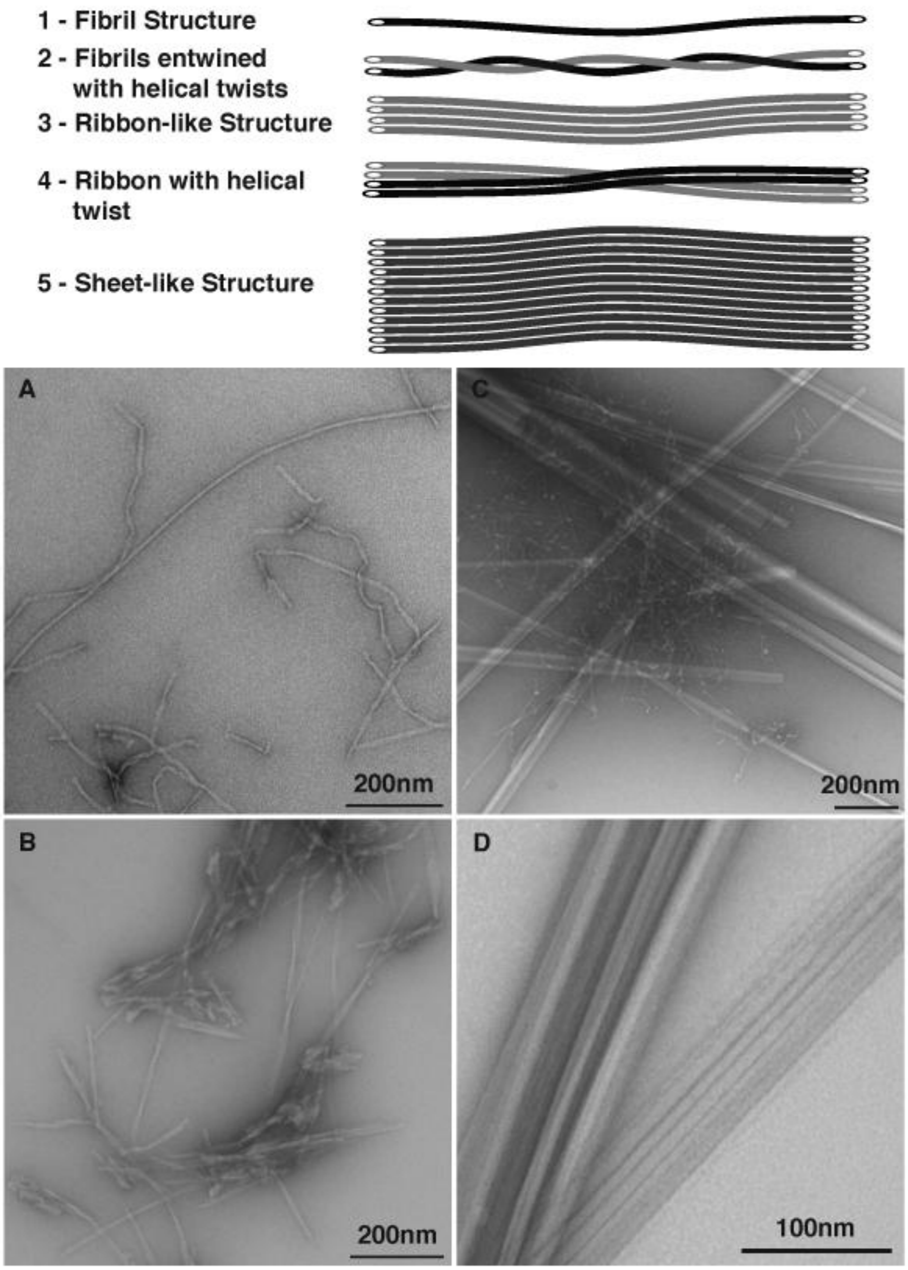

**FIGURE 1.** Fibril formation by human IAPP 1-37 (A), 8-37 (B), and 20-29 (C and D) peptides. Polymorphism of fibrillar forms was clearly evident for 1-37, 8-37, and 20-29. A range of fibrillar structures detailed in the schematic diagram was observed. The 1-37 and 8-37 forms contained a mixture of fibril and ribbon-like structures. The 20-29 form contained a diverse size range of fibrils and sheet-like tubules; at higher resolution, the sheets appear to be formed from cross-linked fibrils in a parallel alignment. The scale bars indicate 200 nm (A–C) or 100 nm (D).

### TEM of Catalase Binding to IAPP Fibrillar Forms

Fibrils were formed from human IAPP 1-37, 8-37, and 20-29, and then incubated with catalase. The 1-37 (Fig. 2A), 8-37 (Figs. 2B and 2C), and 20-29 (Fig. 2D) fibrils all showed binding to catalase. In the 20-29 samples (Fig. 2D), there was also some aggregation of catalase not bound to fibrils, suggesting the ability of IAPP 20-29 to cross-link the catalase tetramers to each other, a feature likely to be caused by smaller IAPP

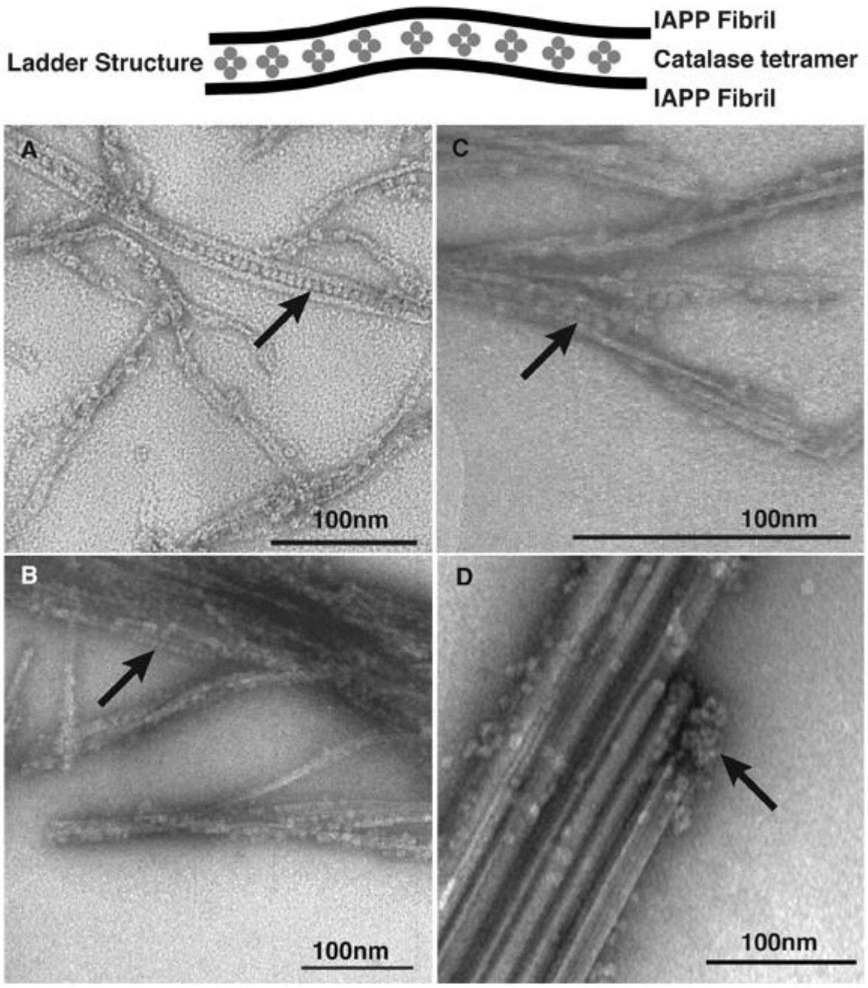

**FIGURE 2**. Catalase binding to human IAPP fibrils — 1-37 (A), 8-37 (B and C), and 20-29 (D) — all showed catalase binding (example indicated by arrow in A–C). There was also cross-linking of two fibrils by a chain of HEC molecules in the 1-37 (A) and 8-37 (C) samples in a manner detailed in the schematic diagram. The HEC aggregates were observed in the 20-29 (D) sample (arrow). The scale bars indicate 100 nm.

aggregates/oligomers, such as dimers, that are below the resolution of the TEM. The IAPP 1-37 and 8-37 samples showed polymorphisms with both fibril and ribbon-like structures, and there was catalase binding to the fibrils primarily with IAPP 1-37 (Fig. 2A), and to both the fibrils and ribbons for IAPP 8-37 (Figs. 2B and 2C). The catalase was able to cross-link both the 1-37 (Fig. 2A) and 8-37 (Fig. 2C) fibrils to form ladder-like structures (as illustrated in the Fig. 2 schematic diagram), suggesting that the catalase tetramer contained at least two IAPP binding sites that were accessible for interaction with the IAPP fibrils.

For comparison, catalase binding to human IAPP 1-37, Aβ 1-42, human PrP 118-135, and PepA fibrils was compared. All peptides showed polymorphic forms. Results showed clear catalase binding to human IAPP 1-37 (Fig. 3A), Aβ 1-42 (Fig. 3B), and human PrP 118-135 (Fig. 3C) fibrils. There was no detectable catalase binding to the PepA fibrils (Fig. 3D).

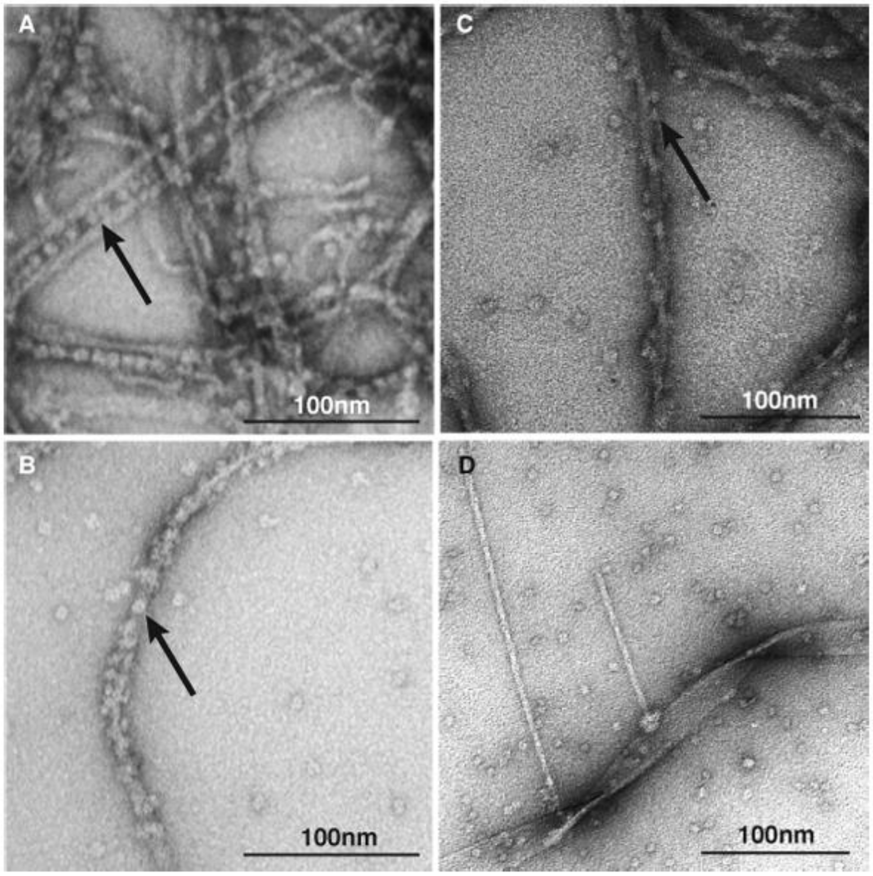

**FIGURE 3**. Comparison of HEC binding to human IAPP 1-37 (A), human Aβ 1-42 (B), human PrP 118-135 (C), and PepA (D) fibrils. The human IAPP 1-37, Aβ 1-42, and PrP 118-135 fibrils all showed binding to catalase (example indicated by arrow in A–C). The PepA showed a polymorphic mixture of fibrils, ribbons, and sheet-like structures, none of which showed catalase binding. The scale bars indicate 100 nm.

### TEM of Displacement Catalase Binding to IAPP and Aβ Fibrillar Forms

Human IAPP 1-37 or Aβ 17-40 fibrils were incubated either with catalase alone or with catalase plus Aβ 31-35. Both human IAPP 1-37 (Fig. 4A) and Aβ 17-40 (Fig. 4C) fibrils bound catalase. When human IAPP 1-37 fibrils were incubated with catalase in the presence of Aβ 31-35 (Fig. 4B), there was no displacement of catalase binding. In contrast, when Aβ 17-40 fibrils were incubated with catalase in the presence of Aβ 31-35 (Fig. 4D), there was significant displacement of catalase from the Aβ fibrils, in agreement with previous observations using Aβ 22-35 fibrils[11]. This suggests that there is either a difference in the binding affinities for IAPP and Aβ fibrils, or that there are differences in the catalase recognition site.

### Immunoassay of Catalase-IAPP Complexes

Samples prepared by incubation of HEC with human and rat IAPP peptides were subjected to immunoassay to identify the presence of catalase-IAPP complexes. Results showed that human IAPP 1-37, rat IAPP 1-37, human IAPP 8-37, and rat IAPP 8-37 all formed complexes that simultaneously bound to both an antiamylin monoclonal (ND1) and anticatalase antibodies (Fig. 5). Complexes between the human

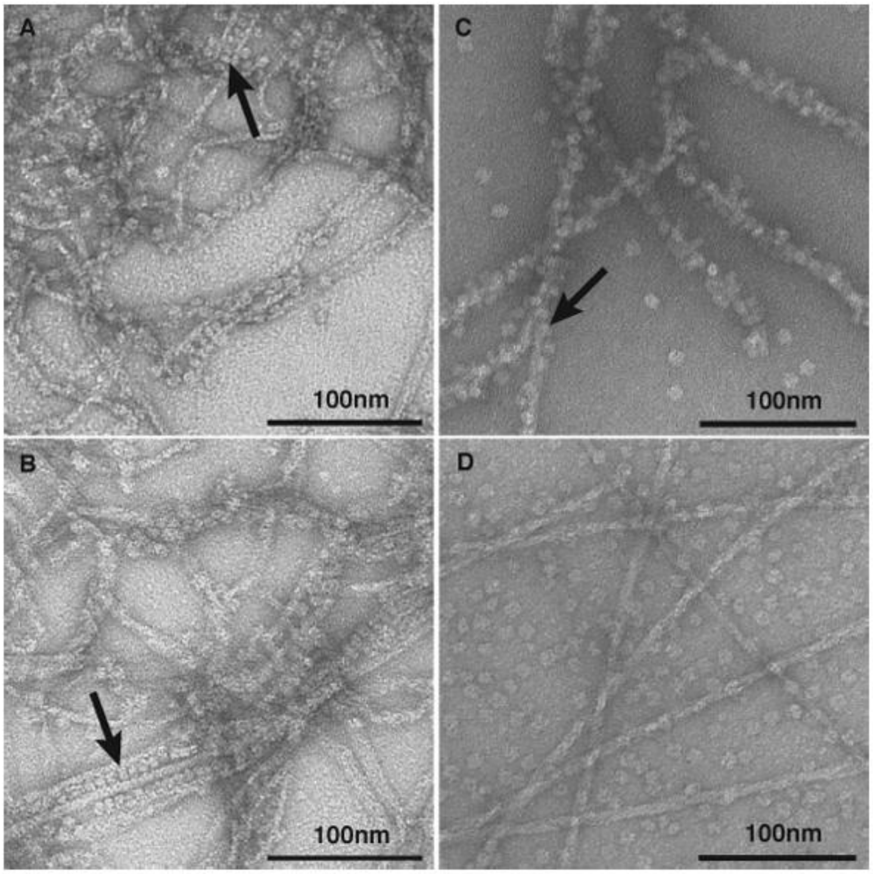

**FIGURE 4.** Effects of Aβ 31-35 fragment on catalase binding to human IAPP 1-37 and Aβ 17-40 fibrils. The human IAPP (A) and Aβ (C) fibrils both bound catalase (example indicated by arrow in A–C). The Aβ 31-35 fragment had no effect on catalase binding to human IAPP (B), but prevented binding to Aβ (D). The scale bars indicate 100 nm.

IAPP 20-29 peptide and catalase could not be detected in this assay due to the absence of IAPP 20-29 binding by this antiamylin antibody.

### Microtiter Plate Binding of IAPP to Catalase

ELISA binding studies were performed using catalase-coated plates and biotinyl IAPP 1-37. The effects of coincubation with a range of unlabeled catalase-binding IAPP, Aβ, and PrP forms were determined. Results showed significant binding of biotinyl IAPP 1-37 to catalase. In the presence of excess unlabeled human IAPP 1-37, 8-37, and 20-29, binding of biotinyl IAPP 1-37 was inhibited (Fig. 6A). Scatchard analysis of binding data demonstrated a $K_D = 0.77 \pm 0.05$ nM (n = 5) for IAPP 1-37 binding to catalase (Fig. 6B). The rat IAPP 1-37 and 8-37 peptides, which have six amino acid differences and are considered nonfibril forming[40,41], also inhibited binding of biotinyl human IAPP to catalase. The Aβ 1-42 and 25-35 peptides also inhibited binding of biotinyl human IAPP to catalase. However the Aβ 31-35, which inhibits Aβ 1-40/42 binding to catalase[11,24], failed to inhibit binding of biotinyl human IAPP to catalase. This confirms the suggestion from the TEM results that showed there is a difference in the ability of Aβ 31-35 to displace catalase binding from IAPP and Aβ fibrils, and that the displacement is specific for Aβ fibrils. The human PrP 118-135 peptide also inhibited binding of biotinyl human IAPP to catalase.

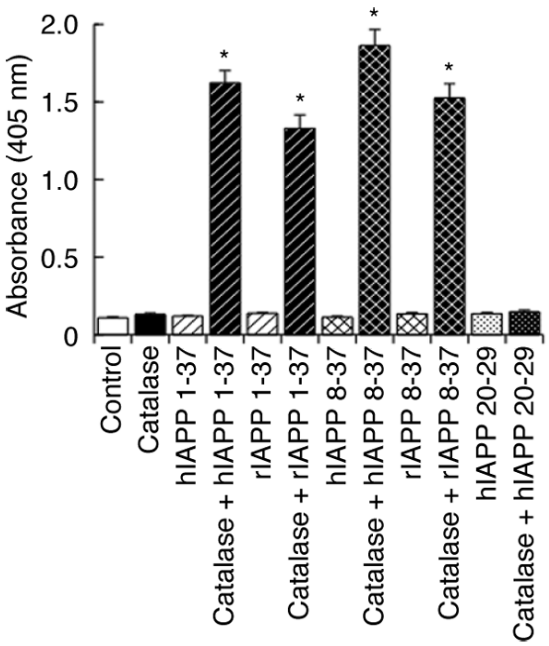

**FIGURE 5.** Immunoassay of IAPP-catalase complexes. Complexes of human IAPP 1-37, human IAPP 8-37, rat IAPP 1-37, or rat IAPP 8-37 with catalase were detected by immunoassay. No complexes between human IAPP 20-29 and catalase were detected due to lack of anti-IAPP binding site in human IAPP 20-29. * = $p < 0.05$ vs. IAPP peptide alone (n = 6; one-way ANOVA).

Using peptide-coated plates and antibody detection of catalase binding, it was shown that HEC bound to human IAPP 1-37 (Fig. 6C). The binding to human IAPP (Fig. 6C) could be inhibited by preincubation of catalase with human IAPP 1-37, rat IAPP 1-37, human IAPP 8-37, rat IAPP 8-37, human IAPP 20-29, Aβ 1-42, Aβ 25-35, or human PrP 118-135. The Aβ 31-35 peptide was unable to inhibit catalase binding to human IAPP–coated plates.

These results suggest a role for the shared Gly-Ala-Ile-Leu–like sequences found in all the peptides capable of inhibiting binding of interactions between human IAPP to catalase.

The streptavidin-alkaline phosphatase polymer conjugate used in all studies to detect biotinyl peptides showed no significant binding to human IAPP–coated plates (streptavidin-alkaline phosphatase polymer 0.052 ± 0.008 vs. buffer control 0.043 ± 0.007). Absorbance values for binding of biotinyl IAPP (2.071 ± 0.137) to human IAPP–coated plates showed significant binding compared to a control (biotinyl-LHRH) peptide (0.172 ± 0.013). Preincubation with monoclonal antiamylin antibody ND1 caused a significant reduction in the absorbance values for biotinyl IAPP binding (0.572 ± 0.031) when compared to biotinyl IAPP plus a control monoclonal (1.897 ± 0.137). These results confirm that the biotinyl IAPP can bind to immobilized IAPP and that this interaction could potentially influence catalase binding. To determine if the inhibition of catalase binding to IAPP was caused by amyloid peptides binding directly to the IAPP, and obscuring the catalase binding site rather than by direct binding to catalase and competitively inhibiting IAPP binding, a series of human IAPP–coated plates were preincubated with human IAPP 1-37, rat IAPP 1-37, human IAPP 8-37, rat IAPP 8-37, human IAPP 20-29, Aβ 1-42, Aβ 25-35, or human PrP 118-135 prior to washing off unbound peptide and addition of catalase. Results showed that none of the peptides tested under these conditions inhibited binding of catalase (Fig. 6D).

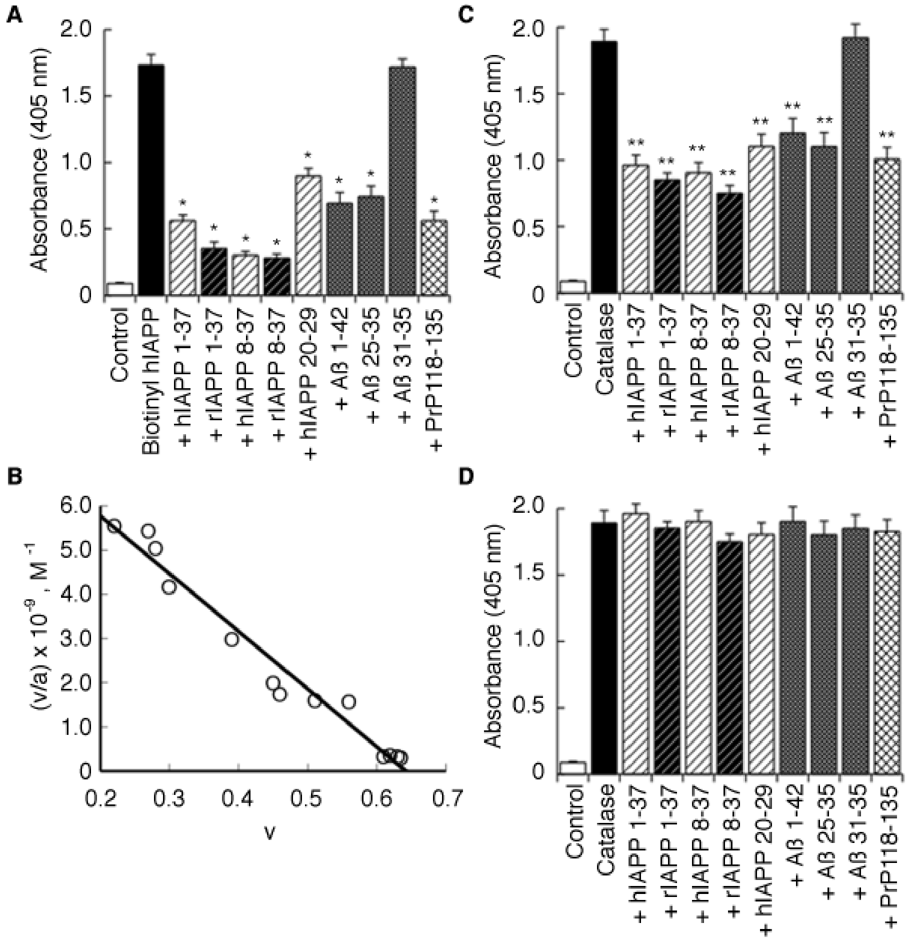

**FIGURE 6**. IAPP binding to catalase. (A) Binding of biotinyl human IAPP 1-37 to immobilized catalase in the presence of unlabeled human IAPP, Aβ, or PrP peptides was inhibited. * = $p < 0.05$ (one-way ANOVA) vs. biotinyl human IAPP 1-37 alone. (B) Scatchard plot of binding of biotinyl human IAPP 1-37 to catalase in the presence of unlabeled human IAPP 1-37. Data were plotted using v against v/a, where v =[($A_0$ - A) / $A_0$]; $A_0$ = absorbance in absence of unlabeled peptide, A = absorbance in presence of unlabeled peptide, and a = concentration of unlabeled peptide added. (C) Catalase binding to human IAPP 1-37–coated ELISA plates in the presence of unlabeled human IAPP, Aβ, or PrP peptides was reduced. ** = $p < 0.05$ (one-way ANOVA) vs. catalase alone. (D) Human IAPP 1-37–coated ELISA plates preincubated in the presence of unlabeled human IAPP, Aβ, or PrP peptides prior to washing and addition of catalase showed no differences in the binding of catalase.

### DISCUSSION

The current study is the first demonstration of a specific interaction between catalase and IAPP. The ability of IAPP and catalase to form immunoreactive complexes (Fig. 5) combined with TEM and microplate demonstration of binding interactions suggests that this is a strong interaction. The affinity constant determined for the reaction was 0.77 nM (Fig. 6B) and this is in a similar range to that observed for Aβ binding to catalase[24]. These microplate studies on the interaction between catalase with IAPP were carried out under conditions that do not favor the formation of fibrillar forms of IAPP[37]; however, the presence of fibrils or other aggregates cannot be excluded. The studies by Higham et al.[42] suggest that IAPP prepared in phosphate buffer forms amorphous aggregates and that fibril formation can be seen by TEM within 3 h; this is within the 4-h incubation times we used for microplate studies and raises the possibility that fibrils could have formed in our samples. The affinity constant is calculated based on the assumption that one molecule of IAPP interacts with one molecule of catalase[38]. The observation that biotinyl IAPP binds unlabeled IAPP suggests that the calculated affinity constant may not be accurate because there were multiple interactions occurring within the wells of the microplate that could have influenced the overall measurement.

The results confirm our previous suggestion[11] that similarities between IAPP and the characterized region of Aβ essential for catalase binding indicate a potential for IAPP binding to catalase. Based on the microplate study plus inhibition of catalase-IAPP interactions by fragments of IAPP, Aβ, and PrP that all contain a Gly-Ala-Ile-Leu–like sequence, the proposed region of IAPP responsible for catalase binding is residues 24-27. This region of IAPP responsible for catalase binding has been suggested to be within a loop or bend region of IAPP that separates two β-sheet components that are central to fibril formation[39]. Molecular modeling has also suggested that residues 22-27 form a β-sheet region that is one of three that form the amyloid core[43]. In either case, the key for catalase binding is the accessibility of this region within the fibril structure. The ability of catalase to bind IAPP 1-37, 8-37, and 20-29 (Fig. 2) suggests that this region is accessible on the surface of the fibrils. The observation that the nonfibril forming rat IAPP peptides can inhibit binding of catalase to labeled human IAPP (Fig. 6A) suggests that the interaction with the 24-27 region does not require it to be part of a fibril. The IAPP 1-37 peptide contains a disulfide bridge between residues 2 and 7 that was intact in our studies. The ability of both IAPP 8-37 and 20-29 to bind catalase suggests that the disulfide bridge region does not play a role in this interaction.

The ability of catalase to bind PrP fibrils provides a further link between amyloid fibrils and catalase. The catalase binding is not shared with PepA, a fibril-forming bacterial peptide[44]. The binding of amyloid fibrils to endogenous extracellular proteins may contribute to their pathological actions. Previous studies have shown that trypsin binds to both Aβ and IAPP[45]; the authors suggested that this action may inhibit the proteolytic activity of the enzyme.

The Aβ binding to catalase is known to inhibit the breakdown of hydrogen peroxide by catalase[24,46,47] and may contribute to oxidative stress associated with Alzheimer’s disease[48]. The links between diseases associated with IAPP and catalase levels raise the possibility that interactions between these two compounds could have pathological consequences, by increasing oxidative stress in either DM[29] or obesity[49]. Further studies on the direct effects of IAPP on the peroxidase activity of catalase should determine whether these interactions have similar effects to those observed when Aβ binds catalase[24].

An interesting difference between the interactions between IAPP and Aβ was the ability of catalase to cross-link IAPP fibrils (Fig. 2A). This suggests that the binding site for IAPP is accessible on more than one of the catalase tetramer components. Such cross-linking was not observed with catalase binding to fibrils formed by a range of Aβ fragments[11], nor to the fibrils formed by the fragment of the human PrP in this study. This may reflect differences in the accessibility of the Gly-Ala-Ile-Leu–like domain on different fibril forms. Polymorphism of Aβ fibril forms influences catalase binding[11] and in this study, polymorphism of IAPP fibril forms is also shown to influence catalase binding. The observation that IAPP fibrils bind catalase more readily than the ribbon-like structures suggests that the IAPP 24-27 region may be on the surface of thin or thick fibrils, but more obscured when the fibrils assemble into the ribbon and sheet-like structures. The differences between the models of Luca et al.[39] and Kajava et al.[43] for IAPP fibril formation reflect differences in the structure adopted by the 22-27 region. These models may represent different polymorphisms of the IAPP fibril. It may well be the case that the structure adopted by the 24-27 region differs between the straight fibril and ribbon-like structures that we (Fig. 1) and Luca et al. observed[39]. With human IAPP 20-29 fibrils, the majority of the binding was on the edges of these tubular and sheet-like polymers of individual fibrils (Fig. 2D). The altered specificity of catalase suggests that this IAPP binding protein could be used to distinguish between different polymorphic forms of IAPP.

The IAPP 20-29 peptide was also able to cross-link catalase tetramers, probably due to the presence of smaller IAPP 20-29 dimers or oligomers that were below the resolution of the TEM (Fig. 2D). Similar cross-linking of catalase by Aβ peptides has previously been reported[11].

The binding of catalase to biotinyl IAPP demonstrated using an ELISA binding assay showed the ability of a range of fibril-forming peptides to inhibit this binding competitively (Fig. 6). The inhibition of binding of catalase to biotinyl IAPP by unlabeled peptides could either be due to the unlabeled peptide binding to catalase or binding directly to the biotinyl IAPP and shielding the recognition site. To exclude the latter possibility, ELISA plates were coated with IAPP 1-37 and incubated with the IAPP, Aβ, or PrP peptides prior to washing to remove unbound peptides and addition of catalase. The results showed that under these conditions, a similar amount of catalase binding was observed (Fig. 6D), suggesting that the unlabeled peptides were not able to shield the IAPP recognition site. The ability of IAPP, Aβ, and PrP peptides to inhibit interactions between biotinyl IAPP and catalase are therefore likely to be due to the unlabeled peptides binding to catalase and competitively inhibiting binding of the labeled peptide. Unlike the well-characterized interactions between Aβ and catalase[11,24], the Aβ 31-35 fragment had no effect on labeled IAPP binding in either the ELISA or in the TEM assays (Fig. 4). This raises the possibility of either a different IAPP recognition site or that the binding to IAPP was of higher affinity than that of Aβ. From previous studies, the binding affinity of Aβ 25-35 for catalase was in the low nanomolar range and appeared to have a single binding site, based on the analysis of the affinity data[24]. The ability of Aβ 25-35 to inhibit the binding of biotinyl IAPP to catalase points to IAPP having a higher affinity rather than a different binding site. The measurement of the affinity constant for IAPP binding to catalase is slightly higher than Aβ, however, methodological limitations to the results obtained as discussed above make it difficult to draw a firm conclusion from this data. The ability of the PrP 118-135 fragment and Aβ to inhibit IAPP binding to catalase points towards the common Gly-Ala-Ile-Leu–like domain playing a role[11].

The known toxicity of fibrillar IAPP forms[14,15] and the failure of the rat IAPP to either aggregate or exhibit neurotoxicity[15,40,41] suggests that the sequence differences contribute to the aggregation and toxicity. Interestingly, two of these differences are within the Gly-Ala-Ile-Leu–like IAPP 24-27 sequence, with a substitution of Ala 25 for Pro and Ile 26 for Val. Since the rat IAPP form inhibits biotinyl human IAPP binding to catalase, it suggests that these substitutions fail to alter the catalase binding site significantly. The Val 26 substitution is much more conservative and with the PrP 118-135 sequence, which also contains a Val at an equivalent position, binding catalase suggests that the change does not influence catalase binding to fibrillar amyloid peptides containing a Gly-Ala-Ile-Leu–like domain.

The formation of IAPP fibrils and the fibril surface structures are influenced by the assay conditions[50]. As such, the artificial conditions employed in this study using TEM and ELISA may not provide an accurate reflection of the interactions under physiological conditions. The elevation of catalase[31] and presence of IAPP containing deposits[1] in the pancreas of Type II DM patients could be a physiological location for such an interaction, and detection of immunoreactive complexes in this tissue would be a step to confirming whether our *in vitro* interactions take place within a physiological *in vivo* setting.

The ability of catalase to interact with both human and rat IAPP raises the possibility that it could also interact with the IAPP agonist Pramlintide, a synthetic IAPP peptide derived from human IAPP, with substitutions of Pro residues at positions 25, 28, and 29 that mimic rat IAPP[51]. The uses of Pramlintide in DM care[52] and as a potential obesity therapeutic[32] raise the possibility that modification of catalase actions could be a side effect. Since oxidative stress is linked to both DM[29] and obesity[53], there is potential for these interactions to have pathological consequences, suggesting that further studies on the interactions between Pramlintide and catalase are required. Pramlintide itself is also potentially amyloidogenic[54,55] and since small aggregates of IAPP 20-29 can cross-link catalase, they may also possess this property.

### CONCLUSIONS

The human IAPP peptides form polymorphic structures that comprise small aggregates, fibrils, and ribbon- and sheet-like structures. The binding of catalase is primarily to IAPP fibrils and less to the IAPP ribbon- and sheet-like structures. The IAPP 20-29 region contains the binding site for catalase. The binding of catalase to labeled human IAPP has an affinity constant of 0.77 nM, and can be displaced by unlabeled human or rat IAPP, Aβ, or PrP peptide fragments. A role for the Gly-Ala-Ile-Leu motif in human IAPP residues 24-27 is suggested. The human IAPP 24-27 region has been considered crucial for amyloid fibril formation[41] and catalase derivatives, such as the Aβ binding peptide R9[11], may therefore be useful in prevention of human IAPP fibrilization.

### ACKNOWLEDGMENTS

The financial support by NeuroDelta Ltd. and a U.K. Department of Trade and Industry Grant (LOT/0311684) is gratefully acknowledged.
